# Supplementary material for: Citrus Bergamia and Cynara Cardunculus Reduce Serum Uric Acid in Individuals with Non-Alcoholic Fatty Liver Disease
Source: Medicina (Kaunas). 2022 Nov 26;58(12):1728. doi: 10.3390/medicina58121728 (PMC9784233; doi:10.3390/medicina58121728)
Supplement: Supplementary file 1 [file medicina-58-01728-s001.zip › medicina-2009849-supplementary.pdf]

Supplemental Table S1. Baseline clinical characteristics of participants who taking the nutraceutical according to the tertiles of serum uric acid at baseline.

Variables	I Tertile (n=15)	II Tertile (n=16)	III Tertile (n=16)	*p* ANOVA	*p* Post-Hoc Analysis
**SUA (mg/dL, range)**	3.8-4.4	4.7-5.3	5.4-6.5		
Age (years)	49±7	53±10	54±9	0.25	-
Weight (Kg)	72±13	81±10	86±11	0.009	I vs II 0.039<br>I vs III 0.002
BMI (Kg/m^2)	28.5±4	29.2±3	29.7±3	0.58	-
WHR	0.89±0.1	1.49±2.1	0.97±0.1	0.33	-
Glucose (mg/dL)	89±7	92±9	95±4	0.045	I vs III 0.013
TC (mg/dL)	189±28	191±39	185±34	0.89	-
TG (mg/dL)	87±40	110±50	107±47	0.31	-
HDL-C (mg/dL)	56±13	51±14	51±10	0.40	-
LDL-C (mg/dL)	115±26	118±36	113±32	0.90	-
AST (IU/L)	19±5	21±6	22±8	0.51	-
ALT (IU/L)	20±11	26±15	25±11	0.34	-
γGT (UI/L)	24±20	26±20	26±14	0.96	-
Creatinine (mg/dL)	0.67±0.1	0.87±0.1	0.89±0.1	<0.001	I vs II <0.001<br>I vs III <0.001
**Prevalence**					
Gender (Male, %)	13	69	81	<0.001	-
Physical activity (%)	53	50	44	0.59	-
Smokers (%)	13	38	31	0.27	-
Obesity (%)	28	37	31	0.16	-
NAFLD S3 (%)	40	69	38	0.16	-
Hypertension (%)	20	25	56	0.034	-
Hyperlipidemia (%)	20	56	60	0.030	-
Antihypertensive drugs (%)	13	19	56	0.009	-
Beta-blockers (%)	7	6	6	0.96	-
Diuretics (%)	0	0	31	0.005	-
Lipid-lowering agents (%)	7	13	31	0.07	-
Antiplatelet agents (%)	7	13	13	0.60	-
Urate-lowering drugs (%)	0	0	0	-	-

Note. BMI = body mass index, WHR = waist to hip ratio, TC = total cholesterol, TG = triglycerides, HDL-C = high density lipoprotein cholesterol, LDL-C = low density lipoprotein cholesterol, AST = aspartate aminotransferase, ALT = alanine aminotransferase, γGT = gamma glutamyltransferase, SUA = serum uric acid.

Supplemental Table S2. Changes in clinical parameters after 6 weeks of participants who taking the nutraceutical according to the tertiles of serum uric acid at baseline.

Variables	I Tertile (n=15)	II Tertile (n=16)	III Tertile (n=16)	*p* ANOVA	*p* Post-Hoc Analysis
**SUA (mg/dL, range)**	3.8-4.4	4.7-5.3	5.4-6.5		
Weight (Kg)	-2.0±2.6	-3.6±1.6	-3.5±2.4	0.12	-
BMI (Kg/m^2)	-0.8±1.0	-1.3±0.6	-1.2±0.8	0.20	-
WHR	0.002±0.04	-0.54±2.1	-0.02±0.04	0.39	-
Glucose (mg/dL)	0.7±7	-1.0±8	-1.4±5	0.65	-
TG (mg/dL)	4±38	10±46	-5±51	0.66	-
HDL-C (mg/dL)	-2.6±6	-3.7±4	-2.9±7	0.84	-
LDL-C (mg/dL)	-8±26	-13±15	-1±15	0.23	-
AST (IU/L)	-1.3±3	-2.4±5	0.2±9	0.52	-
ALT (IU/L)	-0.1±3	-4.9±8	-2.9±4	0.42	-
γGT (UI/L)	-3.5±8	-7.8±11	-6.7±9	0.06	I vs II 0.01
Creatinine (mg/dL)	-0.01±0.08	-0.03±0.05	0.03±0.06	0.05	II vs III 0.01
SUA (%)	5.1±12	-3.0±9	-4.9±16	0.07	I vs III 0.03

Note. BMI = body mass index, WHR = waist to hip ratio, TC = total cholesterol, TG = triglycerides, HDL-C = high density lipoprotein cholesterol, LDL-C = low density lipoprotein cholesterol, AST = aspartate aminotransferase, ALT = alanine aminotransferase, γGT = gamma glutamyltransferase, SUA = serum uric acid.
